# Physical Properties of Cellulose Derivative-Based Edible Films Elaborated with Liposomes Encapsulating Grape Seed Tannins

**DOI:** 10.3390/antiox13080989

**Published:** 2024-08-14

**Authors:** Constanza Vidal, Johana Lopez-Polo, Fernando A. Osorio

**Affiliations:** 1 Department of Food Science and Technology, Technological Faculty, University of Santiago-Chile—USACH, Av. El Belloto 3735, Estación Central, Santiago 9170022, Chile; constanza.vidala@usach.cl; 2 Laboratorio de Biotecnología de los Alimentos, Instituto de Nutrición y Tecnología de los Alimentos, Universidad de Chile, El Líbano 5524, Macul, Santiago 783090, Chile; johana.lopez@inta.uchile.cl

**Keywords:** liposome, biopolymers, bioactive compounds, mechanical properties, wettability

## Abstract

Combined use of edible films (EF) with nanoencapsulation systems could be an effective alternative for improving the films’ physical properties and maintaining bioactive compounds’ stability. This research work focuses on the combined use of EF of cellulose-derived biopolymers enriched with liposomes that encapsulate grape seed tannins and on the subsequent evaluation of the physical properties and wettability. Tannin-containing liposomal suspensions (TLS) showed 570.8 ± 6.0 nm particle size and 99% encapsulation efficiency. In vitro studies showed that the release of tannins from liposomes was slower than that of free tannins, reaching a maximum release of catechin of 0.13 ± 0.01%, epicatechin of 0.57 ± 0.01%, and gallic acid of 3.90 ± 0.001% over a 144 h period. Adding liposomes to biopolymer matrices resulted in significant decrease (*p* < 0.05) of density, surface tension, tensile strength, elongation percentage, and elastic modulus in comparison to the control, obtaining films with greater flexibility and lower breaking strength. Incorporating TLS into EF formulations resulted in partially wetting the hydrophobic surface, reducing adhesion and cohesion compared to EF without liposomes. Results indicate that the presence of liposomes improves films’ physical and wettability properties, causing them to extend and not contract when applied to hydrophobic food surfaces.

## 1. Introduction

Condensed tannins from grape seeds, called proanthocyanidins, are monomeric, oligomeric, or polymeric flavonoids of flavan-3-ol units. The predominant components of these tannins are (+)—catechins (28.2%), (−)—epicatechins (8.8%), and gallic acid (1.3%) [1,2,3]. Grape seed tannins are natural phenolic compounds known for their potent antioxidant activity [4]. These tannins, found in abundance in grape seeds, have been the subject of increasing interest in scientific research due to their multiple health benefits. Among their most notable properties are the ability to neutralize free radicals, reduce oxidative stress, and prevent chronic diseases associated with oxidative damage, such as cardiovascular diseases and certain types of cancer [5,6]. Grape seed tannins are a rich source of proanthocyanidins, which are polymers of flavan-3-ols. These proanthocyanidins are highly effective in scavenging free radicals due to the presence of multiple hydroxyl groups in their molecular structures, which can donate electrons to neutralize free radicals. Studies have shown that grape seed tannins possess significantly higher antioxidant capacity than other natural antioxidant sources, such as vitamins C and E [7,8]. This potent antioxidant activity makes them promising candidates for applications in functional foods and nutraceutical products.

However, their oral administration and use as bioactive compounds in the food industry are limited due to their instability in the gastrointestinal tract, low water solubility, and poor bioavailability [9,10]. An innovative alternative to overcome these limitations of tannin instability is using liposomes as a nanoencapsulation technology, which allows them to be wrapped and protected from external conditions. Liposomes are nanometer-sized spherical vesicles composed of a phospholipid bilayer with hydrophilic heads and hydrophobic fatty acid tails surrounding an aqueous compartment [11]. These liposomes can be used to encapsulate hydrophilic, hydrophobic, and amphiphilic compounds [12]. Encapsulation in liposomes would stabilize and protect tannins against a series of chemical and environmental changes (temperature, pH, oxygen) typically used in the food industry, as well as improve their bioavailability and achieve a controlled release [13].

Recently, the combination of nanoliposomes with edible films (EF) has been studied as a novel strategy to improve and protect the physicochemical characteristics of bioactive compounds, prolong the shelf life of foods with edible films and achieve a gradual release of the compound [14,15]. EF are thin, flexible matrices that are made from natural biopolymers, applied directly to the surface of food [16]. EF are eco-friendly, non-toxic, biodegradable and can be consumed along with the food product [17,18]. Among the most commercially used biopolymers for EF production are polysaccharides derived from cellulose, such as Hydroxypropylmethylcellulose (HPMC) (CAS Number 9004-65-3) and Carboxymethylcellulose (CMC) (CAS Number 9004-32-4), which are generally recognized as safe (GRAS) biocompatible with other water-soluble materials, tasteless, colorless and transparent [19,20].

In the literature, some works have been published on the combined use of various biopolymers for edible coatings such as CMC with nanoliposomes that encapsulate antioxidants, i.e., sour tea extract (*Hibiscus sabdariffa* L.). The results showed that CMC and nanoliposomes from tea extracts reduce the oxidative deterioration process in fried chicken nuggets and generate a softer texture of the product [21]. Other works have also applied edible films and liposomes in meat products such as chicken fillets and cheese slices working with HPMC and к-carrageenan edible films, which improved thermal stability, transparency and mechanical properties. Furthermore, it demonstrated strong antibacterial activity against food-borne Gram-positive and Gram-negative bacteria, which may have potential applications in extending the shelf life of these foods [22].

Although the research that has been developed in terms of the combination of films with liposomes is extensive, knowledge about polyphenols in edible films in the food industry is still in an incipient stage [23]. To the best of our knowledge, no work has been published that delves into the combination of an edible film containing liposomes encapsulating grape seed tannins. Therefore, this research aimed to evaluate the physical, mechanical, and wettability properties of edible films made from cellulose-derived biopolymers, specifically HPMC and CMC, enriched with liposomes encapsulating grape seed tannins. This analysis aims to determine the potential of these films for direct application in foods.

## 2. Materials and Methods

### 2.1. Materials

Grape seed tannins (TANIN VR GRAPE^®^; CAS Number:1401-55-4) were obtained from Laffort Chile (suitable for the products intended for direct human consumption in accordance with Regulation (EU) 2019/934). Soy lecithin was provided by Dimerco (Región Metropolitana, Chile). Citric acid anhydrous, tri-sodium citrate 2 hydrate, ethanol (99.9%), glycerol (>99%), ethyl acetate (99.8%), ethanol (99.9%) were obtained from Winkler (Región Metropolitana, Chile). Hydroxypropylmethylcellulose (HPMC) (Metrocel E19 Grade, Dow Wolff Cellulosics; CAS Number 9004-65-3), Carboxymethylcellulose (CMC) (CAS Number: 9004-32-4) (Sodium salt), (+)—Catequin hydrate, (−)—Epicatechin, Gallic acid (anhydrous), 2,2-diphenyl-1-picryhydrazyl (DPPH^●^), 2,2′-Azino-bis(3-ethylbenzothiazoline-6-sulfonic acid) diammonium salt (ABTS^●+^), Trolox ((±)—6-hydroxy-2,5,7,8-tetramethylchroman-2-carboxylic acid), Methanol (99.9%), Trifluoracetic acid (TFA) (>99%), Acetonitrile (ACN) (99.9%), L-cysteine (98%) and dialysis bag (cellulose membrane of molecular weight 14,000 Da) were obtained from Sigma-Aldrich (St. Louis, MO, USA).

### 2.2. The Extraction of Partial Purified Phosphatidylcholine (PC)

The extraction of partial purified phosphatidylcholine (PC) was done from food-grade raw soy lecithin following the methodology described in our previous work with some modifications [24]. Soy lecithin (10 g) was dissolved in ethyl acetate (50 mL) at 20 °C. Then, distilled water (2 mL) was slowly added with manual stirring. The lower phase was separated by decantation and dispersed with acetone (30 mL), crushed with a glass rod, and the grinding process was repeated with a new aliquot of acetone. The precipitate was filtered under vacuum and dried in a desiccator at 20 °C for 48 h obtaining the partial purified PC. With this acetone extraction and washing method, a purification level of 95% of the lipids present is achieved. Taladrid et al. (2017) supports the effectiveness and reliability of the partial purified PC extraction methodology used in this study [24].

### 2.3. Preparation of Tannin-Containing Liposome Suspensions (TLS)

The condition for liposome preparation was determined based on previous studies conducted in our laboratory, which assessed stability over time and the formation of precipitates. During optimization, variations were made in the pH, partial purified PC, glycerol and bioactive compound concentrations. After evaluating various combinations of these factors, optimal concentrations were selected that provided the best stability and efficacy in tannin encapsulation. These concentrations were utilized in liposome preparation to ensure efficient encapsulation and controlled release of bioactive compounds. The tannin-containing liposome suspensions (TLS) were prepared using the heating/homogenization method with lamellarity reduction with some modifications [2]. Grape seed tannins (1 mg/mL) and partial purified PC (10 mg/mL) were dissolved in ethanol-citrate buffer (0.1 M at pH 3) at 80 °C for 1 h. Then, glycerol (0.76% *w*/*v*) [24] and a second heating period was carried out at 80 °C for 1 h. Then, 5 cycles of vortex and 20 cycles of ultrasound (HIELSCHER UP100H, Heidelberg, Germany, max. 100 W) were applied at 90% amplitude and 22.5 Hz. Two control samples were prepared: Free tannin suspension (FTS), prepared by dissolving tannins in ethanol-citrate buffer, and empty liposome suspension (LS), prepared by dissolving partial purified PC in ethanol-citrate buffer and following the same heating/homogenization procedure as the TLS sample.

### 2.4. Particle Size, Polydispersity Index and Zeta Potential

The analyzes of particle size, polydispersity index and z-potential were carried out via the dynamic light scattering (DLS) technique, using a refractive and phospholipid index of 1.330 and 1.334, respectively, and the measurement angle was 173° Backscatter (Zetasizer Nano ZS, Malvern Instruments Ltd., Malvern, UK). The samples were diluted 100 times in potassium phosphate buffer (PPB) (10 mM, pH 7.4) and vertical cuvettes of 2000 μL capacity were used at 25 °C.

### 2.5. Encapsulation Efficiency

The encapsulation efficiency (% EE) was determined with the centrifugation (Hanil Scientific Inc. Supra R22, Gimpo, Republic of Korea) method at 21,058× *g* for one hour [25]. The samples were quantified using Ultra High Performance Liquid Chromatography (UHPLC) (Thermo Fisher Scientific Ultimate 3000, Waltham, MA, USA) with the tannin standards: catechin, epicatechin and gallic acid, as detailed in the methodology Section “TLS Thiolysis and UHPLC Quantification”. The *EE* % was determined with Equation (1).

(1) $$EE\;\%=\frac{{\left[T\right]}_{E}\;-{\left[T\right]}_{L}}{{\left[T\right]}_{E}}$$

where: [*T*]*_E_* is the initial concentration of tannins in the liposomal suspension; [*T*]*_L_* is the concentration of free tannins in the medium.

### 2.6. Antioxidant Activity Analysis

#### 2.6.1. ABTS^●+^ Radical Scavenging Assay of TLS

The TLS samples were analyzed by the 2,2′-azino-bis (3-ethylbenzothiazoline-6-sulfonic acid) (ABTS^●+^) radical scavenging method. To do this, 20 µL of TLS and 980 µL of ABTS^●+^ were mixed and left for 10 min at 25 °C in the dark [26]. The control sample was prepared with 20 µL of ethanol with 980 µL of ABTS^●+^. The percentage inhibition of ABTS^●+^ was determined at 734 nm using Equation (2).

(2) $${\%\;\mathrm{ABTS}\;}^{●+}{\;}_{\mathrm{inhibition}}=\left[\frac{{\mathrm{Abs}}_{\mathrm{control}}\;-{\;\mathrm{Abs}}_{\mathrm{sample}}}{{\mathrm{Abs}}_{\mathrm{control}}}\right]\;\cdot \;100$$

where: ${\mathrm{Abs}}_{\mathrm{control}}$ is the absorbance of the control and ${\mathrm{Abs}}_{\mathrm{sample}}$ is the absorbance of the TLS sample.

#### 2.6.2. DPPH^●^ Radical Scavenging Assay of TLS

TLS samples were analyzed by the 1,1–diphenyl-2 picrylhydrazyl (DPPH^●^) method. For this, 125 µL of TLS was mixed with 4875 µL of DPPH^●^ and allowed to rest for 30 min at 37 °C in the dark [27,28]. The control sample was prepared with 4875 µL of DPPH^●^ and 125 µL of absolute methanol. The absorbance value of the control solution used was 0.7 ± 0.02. The percentage inhibition of DPPH^●^ was determined at 514 nm using Equation (3).

(3) $${\%\;\mathrm{DPPH}\;}^{●}{\;}_{\mathrm{inhibition}}=\left[\frac{{\mathrm{Abs}}_{\mathrm{control}}\;-{\;\mathrm{Abs}}_{\mathrm{sample}}}{{\mathrm{Abs}}_{\mathrm{control}}}\right]\;\cdot \;100$$

where: ${\mathrm{Abs}}_{\mathrm{control}}$ is the absorbance of the control and ${\mathrm{Abs}}_{\mathrm{sample}}$ is the absorbance of the TLS sample.

### 2.7. Detection of Total Phenolic Compounds (TPC) in TLS

One mL of TLS was added to a reaction mixture containing 1.6 mL of 7.5% sodium carbonate and 200 µL of Folin–Ciocalteu reagent. The reaction mixture was incubated at 25 °C for one hour in the absence of light and the absorbance at 760 nm was determined. The calibration curve was prepared with 5 to 25 (µg/mL) gallic acid.

### 2.8. Microstructure

The microstructure of the TLS was studied using transmission electron microscopy (TEM) with the Talos F200C G2 microscope (Thermo Scientific, Waltham, MA, USA). The samples were negatively stained to enhance contrast and then placed on a copper grid coated with Formvar/Carbon (300 mesh, 3 mm diameter HF 36). Subsequently, a 2% *w*/*v* uranyl acetate solution was added to the samples and allowed to rest for 1 min at 25 °C. Finally, the grids were air-dried in an oven (model LDO-150F, LabTech, Gwangju-si, Republic of Korea) at 25 °C for 30 min.

### 2.9. In Vitro Release of Tannins from Liposomes

The in vitro release of tannins was carried out following a methodology similar to that of Lopez-Polo et al. (2020), with some modifications, and using a simulated medium of ethanol–water fatty foods (50:50) according to EU Regulation No. 10/2011 [29]. The fatty food simulant was chosen for its accuracy in simulating contact conditions with fatty foods, aligning with our research on the future application of dairy derivatives and fatty foods. Additionally, this simulant has been used in other release studies to mimic fatty foods, thus endorsing its suitability for our purpose [30,31]. To do this, the TLS sample (2.5 mL) was incorporated into a dialysis bag (cellulose membrane with a molecular weight cut-off of 14,000 Da). The dialysis bag was immersed in the simulated medium (50 mL) and incubated at 37 °C, with constant shaking for 144 h [29,32]. Samples (2.5 mL) of the external medium were taken after 0, 0.08, 0.5, 1, 4, 8, 24, 48 and 144 h to quantify the concentration of released tannins by UHPLC and an equal volume of fresh medium was added to the system of dialysis. In this in vitro test, the FTS sample was used as a control. Subsequently, the in vitro release was determined through the percentage of cumulative release (%) calculated with Equation (4). Furthermore, during the in vitro release test, antioxidant activity was evaluated by ABTS^●+^ and DPPH^●^ assays, and TPC were quantified. In vitro release and antioxidant activity studies were performed in triplicate.

(4) $$\mathrm{Cumulative}\;\mathrm{Release}\;\left(\%\right)=\left[\frac{\left[T\right]\;\cdot \;\mathrm{factor}\;\mathrm{dilution}\;\cdot \mathrm{VR}}{\left[T\right]\;\mathrm{total}}\right]\;\cdot \;100$$

where: [T] is the initial concentration of tannins (µg/mL) in the liposomal suspension; VR is the volume of release medium (mL); T total is the total of tannins (µg).

#### TLS Thiolysis and UHPLC Quantification

To quantify the concentration of tannins from liposomes during in vitro release, they were depolymerized using acid thiolysis with cysteine, releasing their monomeric units of flavan-3-ol. Given that tannins are complex flavonoid polymers, analytical standards derived from grape seed tannins were employed, primarily composed of catechin (28.2%), epicatechin (8.8%), and gallic acid (1.3%) [2]. These predominant constituents allowed for the establishment of an indirect quantitative relationship regarding the tannin concentration present in the samples. To carry out thiolysis, the supernatant (200 µL) of the TLS sample was taken and mixed with the Cysteine-HCl-Methanol thiolysis medium (200 µL). Then, it was left to incubate for 1 h at 65 °C and the reaction was stopped with deionized water (1 mL). The analytical standards of catechin (5–100 μg/mL, R^2^ = 0.999), epicatechin (10–100 μg/mL, R^2^ = 0.999) and gallic acid (2.5–50 μg/mL, R^2^ = 0.999) were quantified as measurement of tannins using UHPLC (Thermo Scientific Dionex UltiMate 3000). The UHPLC method conditions were based on the similar methodology by Bianchi et al. (2016), using a C18 column (5 μm, 250 × 4.6 mm, Perkin Elmer, Waltham, MA, USA) and a UV detector at 280 nm. The composition of Phase A was 0.1% trifluoroacetic acid (TFA) in deionized water and Phase B was 0.08% TFA in a 4/1 ratio of acetonitrile/deionized water at a mobile phase flow rate of 1 mL/min [33]. The FTS sample was used as a control during the test.

### 2.10. Preparation of Edible Films (EF)

Different biopolymer formulations were prepared using different concentrations of HPMC (2%, 3% and 4% *w*/*v*) and CMC (0.5, 1 and 2% *w*/*v*) according to preliminary tests carried out in our laboratory. The biopolymers were hydrated with deionized water and glycerol was added in different concentrations (20%, 30% and 40% *w*/*w* with respect to the biopolymer), for one hour with stirring at 90 °C until complete dissolution and cooled to 20 °C. Subsequently, one homogenization cycle with an Ultra-Turrax (IKA T18 digital, Guangzhou, China) was applied at 10,000 rpm for 5 min. The resulting mixture was degassed with an ultrasound bath (Elmasonic S30, Augsburg, Germany) for 15 min, applying three ultrasound cycles to remove air bubbles.

### 2.11. Physical Properties of EF

#### Density and Surface Tension

The density (ρ) of EF was measured at 20 °C using a pycnometer. The surface tension (γ) was determined using the pendant drop method according to Skurtys et al. (2011). Images of the drop shape were taken using a high-resolution optical system (Edmund Optics, NJ, USA) and the surface tension was determined from the fundamental Laplace equation. The solution of this equation was obtained with the Matlab 7.14 software (MathWorks, Inc., Natick, MA, USA) [34].

### 2.12. Preparation of Edible Films with Tannin-Containing Liposome Suspensions (EF/TLS)

According to the results obtained from density and surface tension measurements, the optimal concentrations of HPMC and CMC biopolymers for EF were selected, which were for HPMC (4% *w*/*v* HPMC with 33% *w*/*w* glycerol) and CMC (0.8% *w*/*v* CMC with 27% *w*/*w* glycerol). These optimal concentrations of EF formulations remained constant during the characterization of the edible films’ physical, mechanical, and color properties. The optimal concentrations of HPMC and CMC were mixed with different concentrations of TLS to obtain edible films with tannin-containing suspensions (EF/TLS) with the following formulations (50:50), (60:40), (70:30), (80:20), and (90:10). These formulations were mixed using magnetic stirring for 15 min. Subsequently, one homogenization cycle with an Ultra-Turrax (IKA T18 digital, China) was applied at 10,000 rpm for 15 s. The resulting mixture was degassed with an ultrasound bath (Elmasonic S30, Germany) for 15 min, applying three cycles of ultrasound to remove air bubbles. The samples were stored and refrigerated (4 °C) until their measurements for four months.

### 2.13. Physical Properties of EF/TLS

#### Density and Surface Tension of EF/TLS

The density (ρ) and surface tension (γ) of EF/TLS were measured according to methodology described in Section “Density and Surface Tension”.

### 2.14. EF/TLS Wettability Parameters

To determine the parameters associated with wettability, a smooth hydrophobic surface was used as a food simulant [35,36,37]. The hydrophobic surface was selected due to its high roughness and texture, as previously described by Monasterio et al. (2023), who reported surface roughness characteristics obtained through white light interferometry (WLI), utilizing a root mean square (RMS) roughness of 1.126 μm and a peak-to-valley difference of 6.933 μm [23]. This indicates that the hydrophobic surface exhibits high roughness and irregularities, providing relevant information on how films would behave in environments with similar surfaces. Subsequently, the contact angle (CA) was measured using the sessile drop method [38], on said hydrophobic surface, using a high-resolution optical system (Edmund Optics, Barrington, NJ, USA) and ImageJ software Version 1.8.0 (National Institutes of Health, Bethesda, MD, USA). A drop of the samples was deposited on the surface and photographs were taken in less than five seconds. The wettability parameters were calculated: work of adhesion (*W_a_*), work of cohesion (*W_c_*) and the spreading coefficient (*S_eq_*), using Equations (5)–(7).

(5) $$S_{eq}=W_{a}-W_{c}$$

(6) $$W_{a}=\gamma_{L}\;\left(1+Cos\;\theta \right)$$

(7) $$W_{C}=2\;\gamma$$

where: *θ* is the contact angle between the drop axis and the normal at the drop interface and γ is the surface tension of the liquid.

### 2.15. Mechanical Properties of EF/TLS

#### Tensile Test

For the measurement of mechanical properties, pieces of films were formed from the EF/TLS liquid formulations. To do this, the EF were placed on polystyrene plates and dried in an oven (Lab Tech LDO-150F, Republic of Korea) at 25 °C for 24 h and then placed in a desiccator at 58% relative humidity for 48 h. The solid films were cut into pieces of 80 × 25 mm^2^ and the thickness (δ) was measured with a digital micrometer (Mitutoyo Co., Kawasaki shi, Japan). For the traction analysis, a texturometer (Zwick/Roell BDO-FBO.5T5, Ulm, Germany) was used with jaws separation of 60 mm (*L*_0_) and a path speed of 10 mm/s. The tensile stress and strain were recorded, the slope of which corresponds to the elastic modulus (EM). These tensile parameters of tensile strength (TS) and percentage of elongation at break (EAB) were calculated with Equations (8) and (9).

(8) $$TS=\frac{F_{max}}{A_{t}}$$

(9) $$EAB\;\left(\%\right)=\frac{L_{max}-L_{0}}{L_{max}}\;\cdot \;100$$

where: *F_max_* is the force at the elastic limit (N), A_t_ is the cross-sectional area of the sample (mm^2^), *L_max_* is the distance between the jaws at break (mm).

### 2.16. EF/TLS Color Parameters

The films were used to measure the color of the samples with a colorimeter (MiniScan XE Plus, Houston, TX, USA) that allows obtaining the parameters of L*, a* and b*. Subsequently, the total color difference (∆*E*) and the yellowness index (*YI*) were calculated using Equations (10) and (11).

(10) $$\Delta E=\sqrt{{\left(L*-{L*}_{ref}\right)}^{2}+{\left(a*-{a*}_{ref}\right)}^{2}+{\left(b*-b_{ref}\right)}^{2}}$$

(11) $$YI=142.86\;\left(\frac{b*}{L*}\right)$$

### 2.17. Statistical Analyzes

The experimental results were subjected to one-way analysis of variance (ANOVA) and Tukey’s multiple comparison test using GraphPad Prism 8.01 statistical software (GraphPad software Inc. 8.01, San Diego, CA, USA). Statistical significance was statistically defined at a value of (*p* < 0.05). Microsoft Excel was used to calculate means and standard deviations. Figures were created using GraphPad Prism 8.01. To determine the optimal concentrations of EF, a multilevel factorial design was used, measuring the physical properties of density (ρ) and surface tension (*γ*) using the STATGRAPHICS Centurion XVI software (Version 16.2.03).

## 3. Results and Discussion

### 3.1. Particle Size, Polydispersity Index and Zeta Potential

The concentrations used and the heating/homogenization preparation method employed in this work allowed us to obtain liposome suspensions that encapsulate tannins, which were characterized by their physicochemical properties. Table 1 shows the results obtained for the particle size, polydispersity index and zeta potential of TLS, FTS and LS. It can be observed that the TLS presented an average size of 570.8 ± 6.0 nm, while for the LS a size of 126.8 ± 0.2 nm.

These particle sizes are in the nanometer scale (30–1000 nm), which are favorable to achieve higher solubility, bioavailability and controlled release [39,40,41]. Other authors have reported sizes smaller than 309.9 ± 5.90 nm for liposomes prepared from soy phosphatidylcholine that encapsulate tannins from grape seed extracts, using the sonication heating/homogenization method. Sizes of 238.6 nm for liposomes prepared with phospholipids derived from soy and cholesterol, which encapsulate catechins from grape seed extracts using the reverse phase evaporation method were reported [2,42]. Furthermore, Chen et al. (2014) reported liposomes prepared with egg yolk phosphatidylcholine and cholesterol, which encapsulate catechins with larger size from 725.9 ± 23.9 to 1148.0 ± 87.9 nm using the reverse phase method [43]. These differences in particle size could be associated with the type of method and type of phospholipid used in the preparation of the liposomes, which can influence the stability and final size of the liposomes obtained [44]. In this way, the difference in particle size of the liposomes probably depends on the amount of mechanical force applied to the suspension, that is, when using a greater force, the particle size is reduced [45]. Furthermore, differences in liposome particle size have been observed when applying heating/homogenization and sonication, with some variations in the temperature [2], number of cycles and amplitude [23], as well as the buffer and bioactive used [29]. These methodological modifications could have significantly impacted the properties of the liposomes and consequently, the average particle sizes obtained [46]. Additionally, it has been demonstrated that prolonged ultrasound cycles can significantly affect the size of liposomes [47,48]; this could be attributed to the expansion effect of the lipid bilayer during prolonged sonication, thereby increasing the permeability of compounds encapsulated in the liposomes [48].

Significant differences (*p* < 0.05) were obtained between the polydispersity index values of TLS (0.56 ± 0.06) compared to LS (0.27 ± 0.01), indicating that TLS presented a more dispersed particle size distribution and less homogeneity. In previous studies, it has been suggested that this observation could be attributed to the ability of polyphenols to interact with the lipid membranes of liposomes, resulting in changes in their size and shape. These alterations may lead to more significant variability in particle size and contribute to a higher polydispersity index compared to empty liposomes [49].

Zeta potential is a key variable to evaluate the surface charge and determine the physical stability of liposomes. Table 1 shows the zeta potential value which was negatively charged, showing values of −37.9 ± 2.4 mV and −30.1 ± 1.9 mV for TLS and LS, respectively. These values indicate that the suspensions obtained can be considered stable, since there is a sufficient electrical charge to generate repulsion between the vesicles and avoid possible aggregation or fusion phenomena [50,51].

#### 3.1.1. Encapsulation Efficiency (% EE)

The % EE was determined to quantify the ability to encapsulate tannins in liposomes using the heating and homogenization method employed in this work. The % EE values shown in Table 1 indicate that liposomes are efficient in encapsulating tannins (99.8 ± 0.01% for catechin; 99.7 ± 0.04% for epicatechin and 99.6 ± 0.06% for gallic acid); thus, they show a greater capacity to transport and protect these bioactive compounds inside. Other authors have reported % EE results that are much lower than those reported in this work [52,53,54]. The higher % EE values obtained in our work compared to the liposomes of other authors could be attributed to the liposome preparation technique used (heating and homogenization) with ultrasonication cycles, which has been shown to allow more stable liposomes with better encapsulation efficiency to be obtained [55]. Therefore, the application of ultrasound could be used to improve the % EE and the stability of a liposome loaded with the bioactive compound.

#### 3.1.2. Antioxidant Activity and TPC

Table 1 shows the antioxidant activity of TLS, FTS and LS measured with the TPC, ABTS^●+^ and DPPH^●^ assays. The results demonstrated that FTS exhibited the highest total phenols and antioxidant activity, followed by TLS and LS. The TPC values were significantly (*p* < 0.05) higher for FTS (205.8 ± 3.7 GAE µg/mL) compared to TLS (185.6 ± 2.2 GAE µg/mL) and LS (48.4 ± 0.4 GAE µg/mL). The ABTS^●+^ and DPPH^●^ assay revealed significantly (*p* < 0.05) higher values for FTS (ABTS^●+^ = 86.3 ± 0.4% and DPPH^●^ = 74.28 ± 0.3%) compared to TLS (ABTS^●+^ = 60.0 ± 1.8% and DPPH^●^ = 64.58 ± 0.6%) and LS (ABTS^●+^ = 7.1 ± 0.3% and DPPH^●^ = 22.4 ± 0.5%). LS exhibited significantly lower values (*p* < 0.05) for TPC, ABTS^●+^ and DPPH^●^, and TPC parameters compared to TLS. These results of higher total phenols and antioxidant activity of FTS over TLS were anticipated because FTS (1 mg/mL) presents free tannins in suspension contrasting with TLS, which exhibited encapsulated tannins, as indicated by our % EE results, revealing a 99.8 ± 0.01% of catechin, 99.7 ± 0.04% of epicatechin, and 99.6 ± 0.06% of gallic acid. This is due to the fact that, in TLS, the tannins would be linked to active groups of the phospholipids of the liposomes, which reduces their availability to react with the free radicals present in the ABTS^●+^ and DPPH^●^ assay [2,56]. Olatunde et al. (2019) demonstrated that free extracts of ECHE (coconut shell extract), which contain the polyphenols tannic acid and catechin, exhibited higher antioxidant activity than liposomes encapsulating ECHE [57]. This is due to its greater availability to interact with ABTS^●+^ and DPPH^●^ radicals when ECHE is unencapsulated [53]. In addition to this interaction, it is crucial to consider possible losses caused by heating or exposure to light during the encapsulation process [58]. These factors can induce changes in the chemical structure of antioxidants, decreasing their capacity to neutralize free radicals. In contrast, LS (empty liposomes) had lower antioxidant activity than TLS. This decrease could be attributed to the presence of partially purified phosphatidylcholine (PC) used in the assembly of the liposomes, resulting in a limited intrinsic capacity of phospholipids to eliminate free radicals. These findings are consistent with those from previous studies on nanoliposomes loaded with catechin [59], quercetin [60], and curcumin [61].

### 3.2. Microstructure

**Figure 1:**
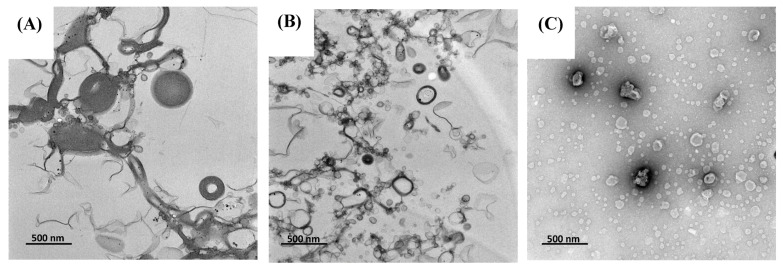
Transmission electron microscopy (TEM) microstructure (**A**,**B**) of TLS and (**C**) LS, magnified 17,500×.

The morphology of TLS and LS were analyzed using transmission electron microscopy (TEM). TEM images revealed that most prepared liposomes exhibited a semi-spherical vesicular and unilamellar structure (Figure 1). In Figure 1A, the TLS sample showed liposome vesicles with variations in their sizes, indicating a heterogeneous distribution. This finding aligns with the polydispersity index (PDI) presented in TLS (0.56 ± 0.06), indicating a broader size distribution. Additionally, the TLS sample (Figure 1B) displayed larger liposome sizes than LS (Figure 1C). These findings are consistent with the results obtained by mean particle size (MPS) analysis, where it was found that the MPS of TLS was larger than that of LS. These results are congruent with those reported for liposomes encapsulating soy protein hydrolysate [62] and liposomes encapsulating naringenin and trans-resveratrol [63], using a combined hydration and ultrasound method. Figure 1 allows for a comparison among treatments by means of their particle size.

### 3.3. In Vitro Release of Tannins from Liposomes

**Figure 2:**
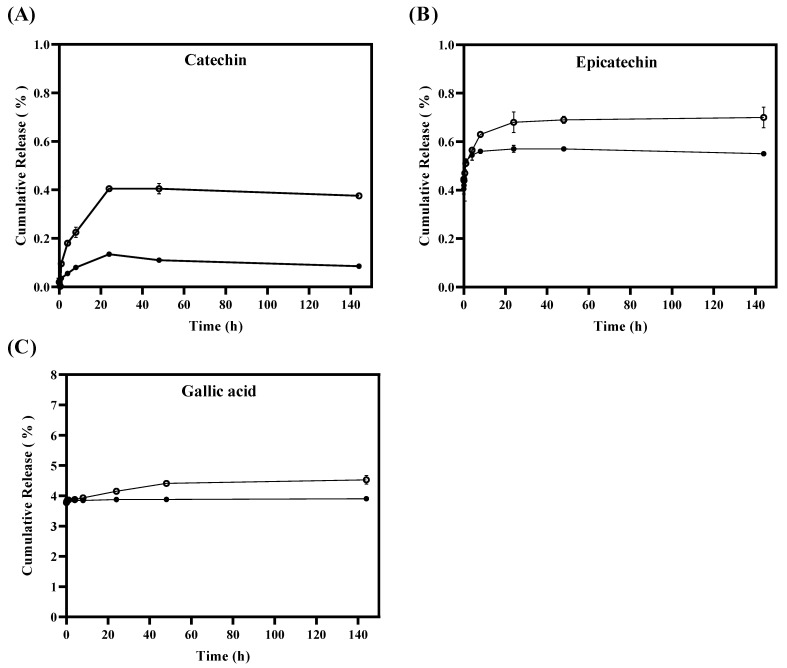
In vitro release of tannins from liposomes TLS (●) and tannins from free suspensions FTS (○) for 144 h by quantifying the cumulative release (%) of (**A**) catechin, (**B**) epicatechin and (**C**) gallic acid.

An in vitro tannin release study was carried out from TLS and FTS using dialysis bag methodology in a simulated fatty food medium for 144 h, with the primary purpose of investigating the stability of tannins and their release from the liposomes. Additionally, the aim was to understand how this release could be affected by environmental factors such as light, temperature, and antioxidant properties. Figure 2A shows that the percentages of cumulative release of catechins released from liposomes are significantly (*p* < 0.05) lower in TLS compared to FTS from the first hour of the in vitro test. The TLS sample reached a maximum release of 0.13 ± 0.01% of catechin after 24 h, maintaining that same release for 144 h, unlike FTS, which reached a maximum release of catechin of 0.41 ± 0.01% after 24 h and remained constant until 144 h. Regarding the releases of epicatechin (Figure 2B) and gallic acid (Figure 2C), they were significantly (*p* < 0.05) lower for TLS compared to FTS after the 24 h of study. It was observed that in TLS a maximum epicatechin release of 0.57 ± 0.01% was reached after 48 h of the study and remained constant for up to 144 h, unlike FTS which reached a maximum epicatechin release of 0.70 ± 0.02% after 144 h. In the case of gallic acid it was 3.90 ± 0.001% after 144 h, unlike FTS whose maximum release was 4.48 ± 0.02% after 144 h. These results indicate that the release rate of tannins encapsulated in liposomes was slower compared to the release rate of free tannins. This suggests that tannins encapsulated in liposomes have a lower release than non-encapsulated tannins. Because the encapsulated tannins would be contained in the liposome membrane, which generates a tortuous path for the passage of the active compound, they cannot be released quickly since they must pass through the entire liposomal bilayer before interacting with the medium abroad. Furthermore, this decrease in the release rate of tannins encapsulated in liposomes can also be attributed to the fact that polyphenol–phospholipid interactions would be occurring through hydrogen bonds [64,65]. The slower release of bioactive compounds, such as polyphenols, may improve their effectiveness as antioxidants by maintaining a constant release of bioactive compounds into the food over a longer period of time, which could extend the shelf life of the food [66]. This behavior is consistent with other studies that have found that liposomes offer greater affinity and protection to antioxidants, resulting in a slower release of these to the medium [67,68,69,70].

**Figure 3:**
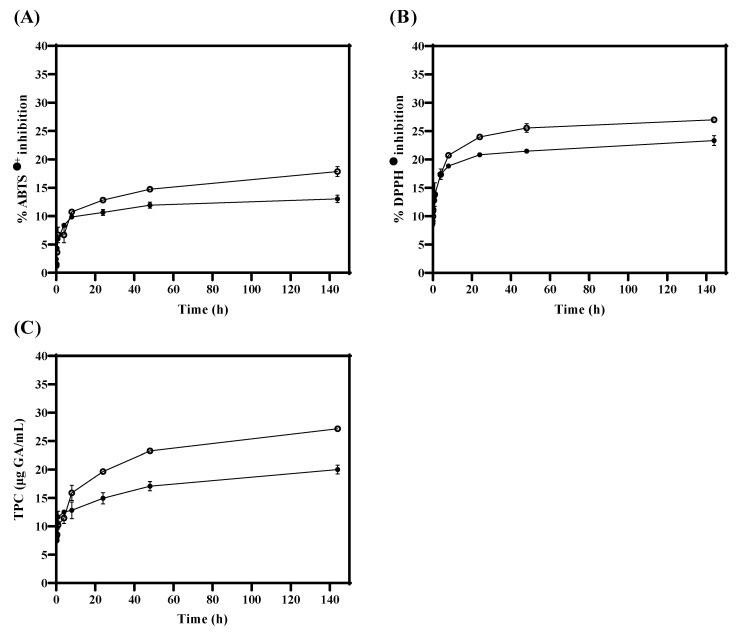
Antioxidant activity and total phenolic compounds of TLS (●) and FTS (○) obtained with the (**A**) ABTS^●+^, (**B**) DPPH^●^ and (**C**) TPC assays during in vitro tannin release.

During the in vitro release test, antioxidant activity (ABTS^●+^ and DPPH^●^) and TPC were measured in TLS compared to FTS. Figure 3A,B show that antioxidant activity values (ABTS^●+^ and DPPH^●^) were significantly (*p* < 0.05) higher in FTS compared to TLS from 24 h. Similarly, in Figure 3C, it was obtained that the quantification of TPC values is significantly (*p* < 0.05) higher in FTS compared to TLS from 8 h. These results suggest that encapsulation of tannins in liposomes could protect antioxidants and maintain their properties in the long term. Therefore, liposomes could be efficient as a protective vesicle against oxidative damage by creating a barrier that separates them from external conditions such as oxygen and/or pH changes [71,72]. Likewise, these results suggest that liposomes could be good nanovehicles for bioactive compounds during digestion since they could increase the bioavailability of antioxidants, by protecting them against the acids that are produced in the chemical digestive process, and therefore could ensure that these reached the absorption process in the intestine [73,74].

Based on the characterization results of the physicochemical parameters of TLS, liposomes with semi-spherical vesicular morphology and nanometric size were observed, which were maintained after the encapsulation of grape seed tannins (% EE of 99.8 ± 0.01% for catechin, 99.7 ± 0.04% for epicatechin, and 99.6 ± 0.06% for gallic acid). Additionally, TLS exhibited lower antioxidant activity than FTS, suggesting greater protection and transport for the bioactive compound. Finally, it was found that TLS showed much slower release than FTS, maintaining its antioxidant properties for 144 h, which could protect the antioxidants and maintain their long-term properties during their food release compared to FTS. Therefore, we have chosen not to use FTS due to its low stability. Instead, we have chosen to work exclusively with TLS for the study of the physical and mechanical properties of the edible films in this study.

### 3.4. Physical Properties of EF/TLS

The TLS were mixed homogeneously with the formulation of the EF, which presented desirable characteristics for their subsequent application in food systems and did not present agglomerations or phase separation over 4 months, as can be observed in Figure 5B. The concentrations of biopolymers used in this study (HPMC and CMC) were selected using a multilevel factorial design that included 18 samples, for which 3 different combinations of biopolymer (2, 3 and 4% *w*/*v*) and glycerol (20, 30 and 40% *w*/*w*) (see Supplemental Table S1) using Statgraphics Centurion XVI software (V16.1.03). To select the optimal concentrations of EF, it was carried out by measuring the density and surface tension with a criterion of maximum values [23], since by increasing the density and surface tension the films could achieve a lower porosity [75] and, therefore, reduce the release values of the bioactive compound [76], which is desired in this work. The optimal concentrations of EF for HPMC were 4% *w*/*v* of HPMC with 33% *w*/*w* of glycerol in its formulation, and for CMC, they were 0.8% *w*/*v* of CMC with 27% *w*/*w* of glycerol. These two EF formulations remained constant, while liposome concentrations varied in different formulations (EF/TLS). Furthermore, to study EF physical properties, we chose to continue our study only with TLS due to the previously described results, which demonstrated better protection of the bioactive compound and slower release compared to FTS, as discussed in Section 3.3. The FTS sample was not used due to its low stability, which ruled it out for application in the physical and mechanical properties of EF/TLS.

### 3.5. Surface Tension and EF/TLS Density

EF/TLS were characterized by their physical properties. Table 2 shows the results obtained for the density and surface tension of the EF. It can be seen that, when incorporating liposomes into the EF formulation, the density values were significantly (*p* < 0.05) lower compared to the control film that did not have liposomes in its formulation. The formulation (70:30) of HPMC with tannin-containing liposome suspensions (HPMC/TLS) and 80:20 of CMC with tannin-containing liposome suspensions (CMC/TLS) reached the lowest density values being 1039.65 ± 1.3 kg/m^3^, and 1014.4 ± 2.1 kg/m^3^, respectively. Ghadermazi et al. (2019) reported that decreasing the density in EF increases the droplet size and apparent viscosity, which can affect the final thickness of the solid EF and reduce their stiffness. These authors suggest that the film density can be controlled and reduced using low-density essential oils to the HPMC suspension [77].

Therefore, lower density values with these EF formulations could reduce the stiffness of the films, allowing better adaptability of the film to the food surface.

Similarly, to the density, the presence of liposomes caused a significant decrease (*p* < 0.05) in surface tension, compared to the control. The 70:30 HPMC/TLS and 60:40 CMC/TLS formulations reached the lowest surface tension values of 39.2 ± 0.5 mN/m and 62.4 ± 0.5 mN/m, respectively. Other authors have shown that reducing surface tension increases wettability and improves the ability of films to adhere to food surfaces, favoring their protection and appearance [18,78,79]. These results are consistent in the literature, where it has been shown that liposomes made with soy lecithin decrease surface tension [80,81]. For example, Jiménez et al. (2014) incorporated soy lecithin nanoliposomes encapsulating orange essential oil and limonene to suspensions of 50:50 sodium caseinate and starch films and observed that the addition of nanoliposomes always decreased the surface tension compared to control [82]. Soy lecithin acts as an amphiphilic surfactant agent, meaning that it has a hydrophilic and a hydrophobic part [83]. When added to an aqueous solution, the lecithin molecules are oriented in such a way that the hydrophilic part is directed towards the water and the hydrophobic part is directed away from it, reducing the surface tension of the suspension, which translates into needing less energy to break the surface [17]. Furthermore, the presence of polyunsaturated fatty acids such as linoleic acid present in soy lecithin can also contribute to the reduction of surface tension [84,85]. Therefore, in this study, the decrease in surface tension could be explained because the liposomes were prepared from phosphatidylcholine that was partially purified from soy lecithin and still contains traces of soy lecithin components, such as polyunsaturated fatty acids in formulations.

### 3.6. EF/TLS Wettability Parameters

**Figure 4:**
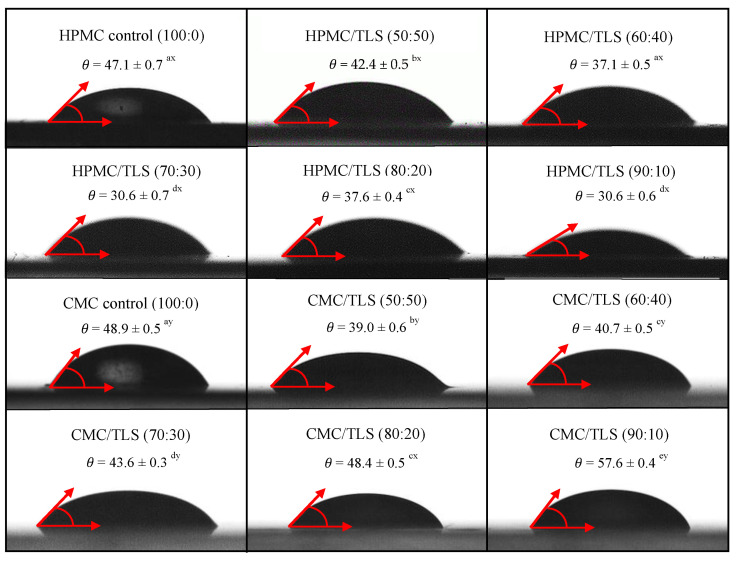
Contact angle between the hydrophobic surface and EF/TLS. (Letters a, b, c, d, and e indicate significant differences in concentrations between EF/TLS. Letters x and y indicate significant differences between HPMC/TLS and CMC/TLS).

Wettability refers to the ability of a liquid to spread over a solid surface and is a key factor to consider in the formulation of EF, since it influences their ability to adhere and cover the surface of the food [86]. The wettability parameters of EF/TLS used in this study were measured by the contact angle method (*θ*), the work of adhesion (*W_a_*), the work of cohesion (*W_c_*) and the spreading coefficient (*S_eq_*) on a hydrophobic surface. The hydrophobic surface selected for the study was utilized for its high roughness and irregular texture, aiming to simulate hydrophobic surfaces resembling those found in fatty dairy products [23]. Figure 4 shows that the contact angles (*θ*) formed between the droplets of the different EF/TLS formulations and the hydrophobic surface are less than 90°, indicating that the EF/TLS formulations moisturize partially the surface (0 < *θ* < 90) [87]. Furthermore, it can be observed that, when incorporating TLS into the EF, the contact angle values are significantly (*p* < 0.05) lower compared to the control without liposomes. These results indicate that the addition of liposomes in the EF formulation causes a significant decrease in the contact angle with the surface, therefore, the liposomes could increase the affinity of the film with the food surface. Similar results were reported by Sapper et al. (2019), who studied soy lecithin liposomes encapsulating thyme essential oil (EO) in starch-gellan (80:20) coatings on the surfaces of apples and persimmons; their study demonstrated that the contact angle values (*θ*) decreased when incorporating the essential oils encapsulated in nanoliposomes in the coating, being lower than 90°, indicating surface wettability and greater extensibility of the coating on the surface of the fruits [88]. Furthermore, it has been reported that the contact angle is a property that also depends on the surface tension of the liquid and generally the contact angle decreases as the surface tension decreases, indicating better wettability of the solid surface by the liquid [17,89,90]. Considering the surface tension values obtained in this work for the EF/TLS formulations and discussed in Section 3.4, it would be reasonable to expect a decrease in the contact angle when incorporating liposomes in the EF, which could improve the adhesion of the films to the hydrophobic surface. These results are positive since they improve and favor the application of these films on different food surfaces.

The wettability of a film on the food surface is determined by an energy balance between Wa and Wc [91]. The Wa corresponds to the energy necessary for the liquid to spread over the surface of the food, while the Wc is a measure of the force of attraction between the molecules of a liquid, which causes contraction of the drop on the surface [38,92]. These two factors are crucial to achieve proper adhesion and prevent film peeling. Table 3 shows that the values of Wa and Wc for EF/TLS decrease significantly (*p* < 0.05) compared to the control in both EF, which indicates that the presence of liposomes in the EF produces a decrease in the values of Wa and Wc on the hydrophobic surface. In the case of HPMC/TLS, the 70:30 formulation presented the lowest values of W_a_ (72.9 ± 1.1 mN/m) and W_c_ (78.3 ± 1.0 mN/m) in relation to the control. While in CMC/TLS, the 90:10 formulation showed the lowest values of W_a_ (105.8 ± 0.4 mN/m) and the 60:40 formulation presented the lowest values of W_c_ (126.1 ± 1.4 mN/m) on the surface.

These results could be favorable for the applications of fatty foods such as dairy derivatives, since they indicate that films with the addition of liposomes easily adhere to surfaces, allowing them to spread and not contract [38]. However, it is crucial to consider the variability among different types of fatty foods and the impact of surface roughness on adhesion [11]. In previous studies it has already been shown that, to obtain better films, a film formulation with low cohesive energy (W_c_) should be selected to obtain better adhesion between the film suspension and the food surface [17,93]. Lopez-Polo et al. (2020) demonstrated that the presence of liposomes and cellulose nanofibers decrease the cohesive work (W_c_) of HPMC, which partially hydrates the almond and chocolate surfaces, since they showed contact angles less than 90° [29]. Therefore, the incorporation of liposomes in the films could improve their adhesion and cohesion capacity and their effectiveness as a protective film, since it would have good adhesion on hydrophobic type surfaces without detaching or sliding. In this sense, our film could have a good application on foods such as dairy products (cheese) and fats in general.

In this study, wettability was also evaluated using the Seq, which indicates the ability of a liquid to spread over a solid surface, with the values closest to zero being the most suitable for coating the surface [94]. Table 3 presents the Seq values of the EF/TLS on the hydrophobic surface. It is observed that for the HPMC/TLS EF, the 70:30 formulation presented the best Seq values, (−5.4 ± 0.1 mN/m), followed by the 90:10 formulation (−5.7 ± 0.1 mN/m), which presented significant differences (*p* < 0.05) compared to the control. In the CMC/TLS case, it was observed that the 50:50 formulations on the food surface presented the best Seq values, (−14.5 ± 0.4 mN/m), followed by the 60:40 formulation (−15.1 ± 0.6 mN/m), which showed significant differences (*p* < 0.05) compared to the control. These results indicate that HPMC/TLS and CMC/TLS formulations have greater spreading and coverage capacity on the food surface compared to formulations that do not contain liposomes. This suggests that liposomes can improve the effectiveness and functionality of films by facilitating better wettability and uniform distribution over the food surface.

### 3.7. Mechanical Properties of EF/TLS

#### Tensile Test

Mechanical properties are used to evaluate the resistance of EFs to maintain and protect foods against physical and mechanical stress during transportation, storage and distribution [95]. In the mechanical properties of the EF/TLS, it is sought that they have adequate flexibility to adapt to the possible deformation of the food during storage without breaking, and, in addition, that they present a high tensile strength and adequate stretch to cover the food [96,97]. Table 4 shows the mechanical properties of the EF/TLS through tensile strength (TS), percentage of elongation at break (EAB) and elastic modulus (EM). The TS refers to the maximum stress supported by the solid films before breaking and EAB indicates the maximum elongation of the solid films before breaking [96]. EM is defined as the ability of a material to resist deformation under tensile stress [98]. In the case of HPMC/TLS, the addition of liposomes caused the values of TS, EAB and EM to decrease significantly (*p* < 0.05) compared to the control.

Furthermore, it was observed that the TS and EAB values were similar for the 70:30 (TS = 17.1 ± 0.1 MPa, EAB = 6.2 ± 0.0%) and 80:20 (TS = 16.7 ± 0.6 MPa, EAB = 6.6) formulations. This indicates that the incorporation of liposomes into HPMC would lead to having lower tensile strength and lower stretch before breakage. Regarding the EM values, it was found that the 70:30 formulation presented significantly (*p* < 0.05) lower values (EM = 6.6 ± 0.3 MPa) compared to the 80:20 formulations (EM = 692.6 ± 0.8 MPa). These results would indicate that the 70:30 formulations would present more flexible characteristics, this being an important quality for the application of films on foods. Our results were in agreement with what was reported by Imran et al. (2012), who studied HPMC films containing nanoliposomes prepared from soy lecithin, which encapsulate nisin, and who observed that the addition of nanoliposomes produced a reduction in interchain links in polymer matrices, and as a result, a decrease in TS (37.0 ± 2.5 MPa) and EM (2228 ± 657 MPa) compared to the control (TS = 59.0 ± 6.8 MPa and EM = 2727 ± 361 MPa) [99]. These results attributed this to the fact that the incorporation of soy lecithin leads to more flexible and less resistant films to breakage [99]. Similarly, De Simone et al. (2020) reported that when adding soy L-α-phosphatidylcholine (PC) liposomes to a HPMC polymer matrix, they found similar TS values of 14.0–16.71 MPa and it was found that the different concentrations of liposomes (1% and 5%) do not significantly affect the particle size and fluidity, but they do present a lower rigidity of the biopolymer [100]. Therefore, the addition of liposomes to edible films could decrease the tensile strength due to their spherical structure and the surface charges of the liposomes that can interact with the functional groups present in the biopolymers of the films, which could affect intermolecular forces by increasing their deformation capacity [101].

Table 4 shows that in the case of CMC/TLS for most formulations, the addition of liposomes significantly decreased (*p* < 0.05) the TS and EM values and presented a significant increase (*p* < 0.05) in EAB values compared to the control. However, the 90:10 formulation presented a significant increase (*p* < 0.05) in the values of TS (19.4 ± 1.6 MPa) and EAB (3.8 ± 0.1%), and a significant decrease (*p* < 0.05) in the values of EM (774.2 ± 0.3 MPa) compared to the control, which would indicate that in this formulation the presence of liposomes showed a positive effect on mechanical resistance, flexibility and with greater rigidity compared to the control without liposomes. Mirzaei-Mohkam et al. (2020) reported that the addition of liposomes prepared with lecithin that encapsulate α-tocopherol (70%) in CMC films significantly decreased (*p* < 0.05) the values of TS (23.1 MPa) and EM (41.2 MPa) compared to the control (TS = 37.0 MPa, EM = 113.9 MPa). While the EAB (52.9%) increased significantly (*p* < 0.05) compared to the control (EAB = 32.3%) [102]. These results were mainly attributed to the structure of lecithin, which can affect the uniformity of the EF, and therefore cause a decrease in the TS and EM values, resulting in softer films [102]. Furthermore, Marín-Peñalver et al. (2019) prepared CMC films containing nanoliposomes loaded with a collagen hydrolysate, prepared with soy phosphatylcholine, and showed that TS (4.2 ± 0.8 MPa) decreases significantly (*p* < 0.05) in the presence of liposomes, when glycerol is added as a plasticizer, and they found an EAB of 50%, which contributes to increasing its elongation and flexibility [103]. Therefore, the behavior of different EF matrices with the addition of nanoliposomes may depend on the nature of the biopolymer, and the most appropriate one can be selected according to the need for protection, release or biological activity of the encapsulated compound.

Our tensile test results indicated that the TS and EM values in the CMC/TLS formulations were significantly (*p* < 0.05) lower compared to HPMC/TLS, indicating that CMC/TLS films are softer and have lower mechanical resistance. Furthermore, CMC/TLS formulations presented EAB values that are significantly (*p* < 0.05) higher compared to HPMC/TLS. Therefore, the results indicate that HPMC EF would have better tensile strength properties and greater stretchability without breaking, which could improve the quality and durability of the final product [104].

### 3.8. EF/TLS Color Parameters

**Figure 5:**
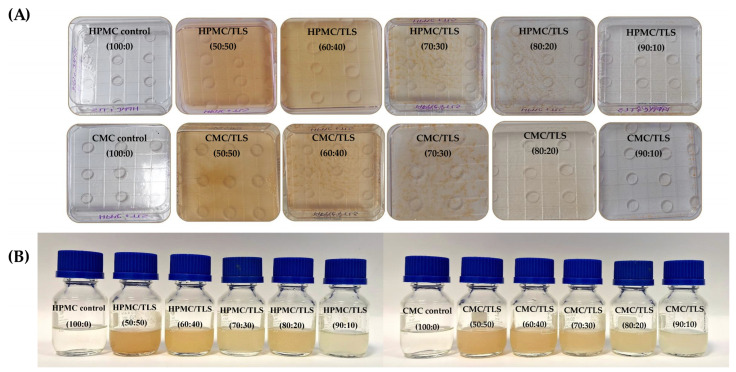
Images of the films produced from EF/TLS (**A**) and the preparation of EF/TLS formulations after 4 months (**B**).

Color is a fundamental characteristic in edible films for food, as it influences consumer perception and acceptability of the final food product. HPMC and CMC correspond to biopolymers that form transparent and colorless films; however, by incorporating specific materials into the formulation, such as glycerol, phospholipids, bioactive compounds, etc., the color properties of the resulting films can be affected [77]. Table 4 shows the results of the total color difference (∆E) and yellowness index (YI) of EF/TLS. When TLS were incorporated into the EF, the ∆E and YI values increased significantly (*p* < 0.05) compared to the control that did not have liposomes in its formulation. The above indicates that there is a perceptible color difference and an increase in yellow color in the samples with liposomes compared to the control sample. However, as there is a lower concentration of TLS in the formulations, the values of ∆E and YI decrease significantly (*p* < 0.05). These results could indicate that, with a lower concentration of TLS, the perceptible color change in the films is reduced, which could be relevant for adjusting formulations according to desired color preferences. In HPMC/TLS, ranges of ∆E (9.5–22.4) and YI (17.0–36.6) values were presented and in the case of CMC/TLS, ranges of ∆E (4.5–21.5) and YI (8.9–35.5) were observed. These colors were in agreement with liposomes prepared with soy lecithin in chitosan/zein films (∆E = 21.4–30.4) [105] and CMC (∆E = 8.38 ± 0.54) [102], as well as the incorporation of sage leaf (SLE) and nettle (NLE) extracts at 15% with HPMC/chitosan films (∆E SLE = 18.3 ± 1.1 and ∆E NLE = 22.2 ± 1.0) [106]. In Figure 5, the different formulations of HPMC/TLS and CMC/TLS can be observed, highlighting a more intense light brown or beige hue in the formulations with higher TLS concentration. Additionally, it is observed that as the concentration of TLS decreases, the coloration becomes more transparent and colorless. These differences in the color of liposome-containing films regarding the control (without liposomes), are due to the fact that the liposomes prepared in this work contain soy phosphatidylcholine which has a light-yellow color, in the same way the grape tannins that were used as encapsulated polyphenol also have a brown color. Therefore, when incorporating these into the EF, their color properties are modified. These findings underscore the importance of assessing how the use of liposomes affects the color properties in edible films; this could have significant implications in ingredient selection and formulation optimization to ensure the acceptability of the product.

## 4. Conclusions

In this research work, it was possible to obtain liposomes that are stable, encapsulate grape seed tannins with a nanometric size (570.8 ± 6.0 nm), and that demonstrate a high encapsulation efficiency of the active compound (99%), maintaining a semi-spherical structure. The values obtained for the analyses of antioxidant activity by ABTS^●+^ and DPPH^●^ were lower for tannins in liposomes, which suggests that encapsulation protects tannins and prevents them from reacting with free radicals; therefore, encapsulation in liposomes could protect antioxidants against external conditions (O_2_, pH, temperature, etc.). Furthermore, we also found that the in vitro release rate of tannins from liposomes was slower than free tannins during the 144 h study, indicating that encapsulation in liposomes could improve the antioxidant efficacy of tannins for a longer period. This would help extend the shelf life of the foods on which the suspensions are applied. The suspensions of tannins encapsulated in liposomes were successfully added to the formulation of edible films, and no phase separation or agglomerations occurred for 4 months. Through studies of the wettability properties of the films, it was found that the addition of liposomes to the formulation caused a significant decrease in density, surface tension and contact angle values; this means that the liposome film partially moistens the hydrophobic surface studied, causing it to spread and not contract when applied, which could favor the protection and appearance of hydrophobic foods on which the film is applied.

## Figures and Tables

**Table 1:** Physicochemical properties of TLS, FTS and LS.

Analysis	TLS	FTS	LS
Mean particle size (nm)	570.8 ± 6.0 ^a^		126.8 ± 0.2 ^b^
Polydispersity index	0.56 ± 0.06 ^a^		0.27 ± 0.01 ^b^
Zeta potential (mV)	−37.9 ± 2.4 ^a^		−30.1 ± 1.9 ^b^
Encapsulation efficiency (% EE)			
(+)—Catechin (−)—Epicatechin Gallic acid	99.8 ± 0.01 ^a^		
	99.7 ± 0.04 ^a^		
	99.6 ± 0.06 ^b^		
Antioxidant activity			
% ABTS^●+^ inhibition	60.0 ± 1.8 ^a^	86.3 ± 0.4 ^b^	7.1 ± 0.3 ^c^
% DPPH^●^ inhibition	64.58 ± 0.6 ^a^	74.28 ± 0.3 ^b^	22.4 ± 0.5 ^c^
TPC GAE (µg/mL)	185.6 ± 2.2 ^a^	205.8 ± 3.7 ^b^	48.4 ± 0.4 ^c^

TLS: Suspension of liposomes containing tannins. FTS: Free tannin suspension. LS: Empty liposome suspension. Liposomes suspensions without tannins. ABTS^●+^: Percentage inhibition of 2,2′-azino-bis (3-ethylbenzothiazoline-6-sulfonic acid) radicals. DPPH^●^: Percentage inhibition of 1,1-diphenyl-2 picrylhydrazil. TPC: Total phenolic compounds. The mean of three replicates is shown. Letters a, b, c indicate significant differences between TLS, FTS, and LS.

**Table 2:** Values of density and surface tension of EF/TLS.

Sample EF/TLS	ρ (kg/m^3^) at 20 °C		γ (mN/m)	
	HPMC/TLS	CMC/TLS	HPMC/TLS	CMC/TLS
Control (100:0)	1078.8 ± 0.7 ^ax^	1042.1 ± 0.6 ^ay^	47.9 ± 0.7 ^ax^	74.5 ± 0.4 ^ay^
(50:50)	1040.0 ± 1.4 ^bx^	1019.4 ± 0.8 ^by^	46.2 ± 0.5 ^abx^	65.1 ± 1.4 ^bcy^
(60:40)	1047.6 ± 1.3 ^bcx^	1023.5 ± 0.7 ^bcy^	42.4 ± 1.0 ^bcx^	62.4 ± 1.2 ^by^
(70:30)	1039.6 ± 1.3 ^bx^	1027.5 ± 0.8 ^cy^	39.2 ± 0.5 ^cx^	64.5 ± 0.2 ^by^
(80:20)	1052.0 ± 1.4 ^cx^	1014.4 ± 2.1 ^dy^	43.5 ± 1.9 ^bx^	68.0 ± 1.5 ^cy^
(90:10)	1049.2 ± 4.7 ^cx^	1034.9 ± 4.7 ^ey^	41.0 ± 0.7 ^bcx^	68.1 ± 1.3 ^cy^

EF: Edible films. EF/TLS: Edible films with tannin-containing liposome suspensions. HPMC/TLS: Hydroxypropylmethylcellulose (HPMC) with tannin-containing liposome suspensions. CMC/TLS: Carboxymethylcellulose (CMC) with tannin-containing liposome suspensions. ρ: density. *γ*: surface tension. The mean of three replicates is shown. Letters a, b, c, d, and e indicate significant concentration differences between EF/TLS. Letters x and y indicate significant differences between HPMC/TLS and CMC/TLS.

**Table 3:** Wettability parameters on hydrophobic surface of EF/TLS.

Sample EF/TLS	Wa (mN/m)		Wc (mN/m)		Seq (mN/m)	
	HPMC/TLS	CMC/TLS	HPMC/TLS	CMC/TLS	HPMC/TLS	CMC/TLS
Control (100:0)	80.5 ± 0.3 ^ax^	123.4 ± 0.7 ^ay^	95.7 ± 0.7 ^ax^	148.9 ± 0.9 ^ay^	−15.3 ± 0.0 ^ax^	−25.6 ± 0.3 ^ay^
(50:50)	80.3 ± 0.8 ^ax^	117.1 ± 0.8 ^by^	92.4 ± 0.9 ^ax^	131.9 ± 0.4 ^by^	−12.1 ± 0.2 ^bx^	−14.5 ± 0.4 ^by^
(60:40)	76.2 ± 1.8 ^abx^	110.8 ± 0.9 ^cy^	84.8 ± 2.0 ^bx^	126.1 ± 1.4 ^cy^	−8.6 ± 0.3 ^cx^	−15.1 ± 0.6 ^by^
(70:30)	72.9 ± 1.1 ^bx^	111.2 ± 0.2 ^cy^	78.3 ± 1.0 ^cx^	129.0 ± 0.5 ^dy^	−5.4 ± 0.1 ^dx^	−17.8± 0.2 ^cy^
(80:20)	77.9 ± 3.6 ^abx^	114.5 ± 0.9 ^dy^	86.9 ± 3.9 ^abx^	137.6 ± 0.7 ^ey^	−9.0 ± 0.3 ^cx^	−22.9 ± 0.4 ^dy^
(90:10)	76.2 ± 0.1 ^abx^	105.8 ± 0.4 ^ey^	81.9 ± 1.4 ^bcx^	137.8 ± 0.2 ^ey^	−5.7 ± 0.1 ^dx^	−31.6 ± 0.7 ^ey^

Wa: Adhesion work. Wc: Cohesion work. Seq: Spreading coefficient. The mean of three replicates is shown. Letters a, b, c, d, and e indicate significant concentration differences between EF/TLS. Letters x and y indicate significant differences between HPMC/TLS and CMC/TLS.

**Table 4:** Mechanical properties and color of EF/TLS.

EF	Sample	TS (MPa)	EAB (%)	EM (MPa)	Δ E	YI
HPMC/TLS	Control (100:0)	22.0 ± 1.0 ^ax^	8.4 ± 0.0 ^ax^	1002.0 ± 0.5 ^ax^	-	3.1 ± 0.5 ^ax^
	(50:50)	6.3 ± 0.1 ^bx^	2.9 ± 0.1 ^bx^	261.4 ± 0.5 ^bx^	22.4 ± 1.2 ^ax^	36.6 ± 1.8 ^bx^
	(60:40)	10.7 ± 0.2 ^cx^	3.8 ± 0.1 ^bx^	540.3 ± 0.9 ^cx^	18.8 ± 0.9 ^bx^	30.7 ± 1.2 ^cx^
	(70:30)	17.1 ± 0.1 ^ax^	6.2 ± 0.0 ^cx^	692.6 ± 0.8 ^dx^	13.1 ± 0.9 ^cx^	22.0 ± 1.3 ^dx^
	(80:20)	16.7 ± 0.6 ^ax^	6.6 ± 0.3 ^cx^	762.9 ± 0.9 ^ex^	14.2 ± 0.3 ^cx^	23.8 ± 0.4 ^dx^
	(90:10)	26.7 ± 0.0 ^dx^	8.7 ± 0.0 ^ax^	953.7 ± 0.3 ^fx^	9.5 ± 0.7 ^dx^	17.0 ± 1.0 ^ex^
CMC/TLS	Control (100:0)	19.0 ± 0.8 ^ax^	2.1 ± 0.1 ^ay^	891.3 ± 2.7 ^ay^	-	2.5 ± 0.2 ^ax^
	(50:50)	0.9 ± 0.5 ^bx^	25.4 ± 1.1 ^by^	0.10 ± 0.0 ^by^	21.5 ± 0.4 ^ax^	35.5 ± 0.8 ^bx^
	(60:40)	1.6 ± 0.4 ^by^	24.9 ± 0.7 ^by^	0.12 ± 0.0 ^by^	20.5 ± 0.9 ^ay^	33.1 ± 1.0 ^cy^
	(70:30)	14.9 ± 1.9 ^abx^	16.5 ± 1.0 ^cy^	24.2 ± 0.0 ^cy^	11.3 ± 0.4 ^by^	19.4 ± 0.5 ^dy^
	(80:20)	7.6 ± 0.0 ^by^	11.8 ± 0.2 ^dy^	111.7 ± 0.3 ^dy^	9.2 ± 0.5 ^cy^	16.0 ± 0.7 ^ey^
	(90:10)	19.4 ± 1.6 ^bx^	3.8 ± 0.1 ^ey^	774.2 ± 0.3 ^ey^	4.5 ± 0.3 ^dy^	8.9 ± 0.5 ^fy^

TS: Tensile strength. EAB: Percentage elongation at break. EM: Modulus of elasticity. ∆E: Total color difference. YI: Yellowness index. The mean of three replicates is shown. Letters a, b, c, d, e, and f indicate significant differences between EF/TLS concentrations. Letters x and y indicate significant differences between HPMC/TLS and CMC/TLS.

## Data Availability

Data are available upon request.

## Supplementary Materials

The following supporting information can be downloaded at: https://www.mdpi.com/article/10.3390/antiox13080989/s1, Table S1. Experimental design for density and surface tension of EF of HPMC/Gly and CMC/Gly at different concentration.
