# Supplementary material for: Physical Properties of Cellulose Derivative-Based Edible Films Elaborated with Liposomes Encapsulating Grape Seed Tannins
Source: Antioxidants (Basel). 2024 Aug 14;13(8):989. doi: 10.3390/antiox13080989 (PMC11351243; doi:10.3390/antiox13080989)
Supplement: Supplementary file 1 [file antioxidants-13-00989-s001.zip › antioxidants-3121509-supplementary.pdf]

Supplementary material - Table S1

Table S1. Experimental design of density and surface tension of the EF.

Sample (N°)	EF (%w/v)		Gly (%w/w with respect to EF)	ρ (kg/m^3) a 20°C		γ (mN/m)	
	HPMC	CMC		HPMC/Gly	CMC/Gly	HPMC/Gly	CMC/Gly
1	4	2	30	1114.2 ± 4.9^{a}	1050.0±1.4^{ab}	55.98 ± 0.26^{a}	64.69±0.91^{a}
2	3	1	20	1101.8 ± 10.2^{a}	1038.0±1.4^{b}	71.11 ±0.23^{b}	74.84±0.36^{b}
3	3	1	40	1038.2 ± 1.4^{c}	1059.0±2.8^{a}	72.07 ±0.75^{b}	65.63 ±0.31^{a}
4	4	2	20	1030.8 ± 6.3^{c}	1051.8±0.3^{ab}	51.31 ±2.92^{a}	62.92±0.53^{a}
5	2	0.5	20	1073.9 ± 2.8^{b}	1049.1±0.6^{ab}	23.65 ±4.48^{d}	76.45± 1.46^{b}
6	4	2	40	1116.3 ± 0.4^{a}	1060.0±2.8^{ab}	57.21 ± 0.54^{a}	74.72 ±1.14^{b}
7	2	0.5	30	1101.2 ± 0.7^{a}	1050.0±1.4^{ab}	54.29 ±0.77^{a}	81.77±0.61^{c}
8	3	1	30	1049.1 ± 1.4^{c}	1038.0±1.4^{a}	65.48 ± 0.42^{c}	83.86±0.52^{c}
9	2	0.5	40	1047.2 ±1.3^{c}	1059.0±2.8^{ac}	67.45 ± 0.31^{bc}	81.65±0.12^{c}

EF: Edible film. ρ: Density. γ: Surface tension. HPMC: Hydroxypropylmethylcellulose. Gly: Glycerol. CMC: Carboxymethylcellulose. The mean of three replicates is shown. Letters a, b, ab and c indicate significant differences between HPMC/Gly, and CMC/Gly concentrations.
